# A case of severe neonatal transient hyperinsulinemic hypoglycaemia without identifiable risk factors: a case report

**DOI:** 10.1186/s12884-022-04763-3

**Published:** 2022-05-19

**Authors:** Asami Osada, Takeshi Arimitsu, Moe Kusakawa, Takane Kin, Mariko Hida

**Affiliations:** Department of Pediatrics, Keio University School of Medicine, 35-Shinomachi, Shinjuku-ku, Tokyo, To 160-8582 Japan

**Keywords:** Gestational diabetes mellitus, Neonatal hypoglycaemia, Hyperinsulinemic hypoglycaemia

## Abstract

**Background:**

Neonatal hypoglycaemia is one of the major metabolic disorders that causes irreversible brain injury. Assessing for maternal glucose metabolism disorders can predict and avoid this perinatal complication. Accordingly, diagnosing maternal gestational diabetes mellitus (GDM) is important in protecting neonatal neurological prognosis. However, there are various methods of screening for maternal GDM. The intervention for neonatal hypoglycaemia also varies within each guideline.

**Case presentation:**

A female infant was born at 37 weeks of gestation by vaginal delivery with no asphyxia. Her mother had no abnormal findings, including glucose metabolism disorders, upon periodic prenatal visits. Upon routine examination at the first hour of life, the baby was lethargic, pale, hypotonic, and rarely cried. An emergent systemic evaluation was performed, and she was diagnosed with severe hyperinsulinemic hypoglycaemia with blood glucose of 11 mg/dL and insulin of 2.7 µU/mL. She was soon fed with milk and her symptoms of hypoglycaemia was resolved before receiving intravenous glucose infusion. Her blood glucose level reached 78 mg/dL 3 h after delivery. She was discharged home with her mother on day 6 of age without relapse of hypoglycaemia. Upon review, we determined that the mother was diagnosed with GDM during her previous pregnancy but not during this current pregnancy. The infant had no developmental delay upon check-up at 6 months.

**Conclusions:**

The infant of this case was not a candidate for neonatal hypoglycaemia screening since her mother had no identifiable risk factors. This case suggests that previous maternal history of GDM might be the cause of neonatal hyperinsulinemic hypoglycaemia. Clinicians need to be aware of the possibility of hypoglycaemia among newborns with a maternal previous history of GDM, regardless of the mother’s current diagnosis. Immediate oral feeding can be one of the treatments, even for symptomatic neonatal severe hypoglycaemia, when rapid intravenous access is difficult. The present case also suggests the necessity of considering neonatal outcomes as well as maternal ones when screening for maternal disorders of glucose metabolism.

## Background

Neonatal hypoglycaemia is a major complication associated with irreversible brain damage. Assessing maternal glucose tolerance during pregnancy can predict and prevent hypoglycaemia soon after birth in infants. Therefore, diagnosing maternal gestational diabetes mellitus (GDM) has a positive influence on neonatal neuroprotection. However, there is no worldwide consensus on the diagnostic criteria for GDM, whose occurrence varies from 6 to 25% depending on each diagnostic approach [1]. Standardized GDM diagnostic criteria are required to avoid overlooking cases of GDM in the clinical setting and to reduce perinatal complications for both mothers and neonates.

The association between neonatal hypoglycaemia, brain injury, and neurodevelopmental disorders has been documented [2, 3]. The severity of hypoglycaemia is key in determining the neurodevelopmental prognosis in children with hyperinsulinemic hypoglycaemia [4, 5]. Although a universal definition of neonatal hypoglycaemia has not been established, guidelines define blood glucose ≤ 40–50 mg/dL as hypoglycaemia [6, 7]. The Pediatric Endocrine Society (PES) recommends that symptomatic hypoglycaemia be treated with intravenous dextrose infusion at any glucose level, and the American Academy of Pediatrics (AAP) recommends intervention when blood glucose levels are below 40 mg/dL.

Here, we report the case of an infant with severe symptomatic transient hyperinsulinemic hypoglycaemia born to a mother who was not diagnosed with GDM or other disorders of glucose metabolism. We successfully diagnosed the infant with hypoglycaemia, based on her symptoms, and immediately started bottle-feeding with formula. Although the mother was not diagnosed with GDM during her current pregnancy, she had a history of GDM during her previous pregnancy, which could be considered as a risk factor for neonatal hypoglycaemia for the present case.

## Case presentation

A 44-year-old woman with one child conceived a second child naturally. The patient had a prior history of GDM; however, her medical history was otherwise unremarkable. During her most recent pregnancy, she was evaluated at periodic prenatal visits. At 24 weeks gestational age, her 50-g glucose challenge test (GCT) at 1 h was 133 mg/dL (cut-off for GDM diagnosis ≤ 140 mg/dL at the study site), and haemoglobin A1c was 5.6 g/dL. She had no perinatal complications, and other glucose metabolism disorders were not detected.

The woman gave birth to a female infant at 37 weeks of gestation by vaginal delivery. The infusion fluids administered to this mother during delivery contained 5% glucose at most. The neonate’s length was 48.5 cm (71.6^th^ percentile, + 0.57 standard deviations [SD]); weight, 3086 g (89.7^th^ percentile, + 1.26 SD); and head circumference, 33.0 cm (59.1^th^ percentile, + 0.23 SD). She was classified as an appropriate-for-gestational age infant. The neonate’s APGAR scores at 1 and 5 min were 9 and 10, respectively, and no interventions were required other than routine care. Her umbilical artery pH was 7.360, and base excess was -0.7 mEq/L. On examination 1 h after delivery, the baby was lethargic, pale, hypotonic, and rarely cried. Considering her clinical features, an emergent systemic evaluation was performed, revealing a blood glucose level of 11 mg/dL and an insulin level of 2.7 µU/mL (> 1.0 µU/mL [8]). While preparing for a glucose infusion, she was fed 10 mL of formula milk for the first time at 1 h of age. Her mother was not ready for breastfeeding, hence, formula feeding was preceded. The baby’s clinical condition immediately improved, and she became awake and active. Following enteral feeding, an intravenous glucose infusion was started (glucose infusion rate of 3.3 mg/kg/min). Her blood glucose level increased to 78 mg/dL 3 h after delivery. The hypoglycaemia did not recur after the intravenous infusion was stopped the next day. As the mother was not ready for exclusive breastfeeding, considering her condition, the baby began breastfeeding on the 2^nd^ day of life and was discharged on the 6^th^ day. She had no developmental delays at the 6-month check-up. Informed consent was documented and obtained from the mother for the anonymized publication of this patient’s details.

## Discussion and conclusions

We diagnosed severe symptomatic neonatal hypoglycaemia in an infant who had no known risk factors for the condition and, therefore, was not a candidate for neonatal hypoglycaemia screening. Regardless of its nonspecific symptoms, the newborn was carefully examined, leading to the diagnosis. Although the mother did not meet the criteria for GDM, medical records revealed that she was diagnosed with GDM during her previous pregnancy 3 years before the present case. The woman’s other child had transient hypoglycaemia in the neonatal period (with a blood glucose level of 45 mg/dL, 1 h after birth). We assumed that GDM during the previous pregnancy was a risk factor for neonatal hypoglycaemia in the present case. This case highlights that the current GDM screening protocols might not identify women at higher risk of delivering babies with hypoglycaemia that require intervention and routine glucose testing. This case also proposes prompt feeding for symptomatic hypoglycaemic infants regardless of its severity, especially if immediate access for intravenous infusion is difficult to achieve.

It is known that women with a prior history of GDM have a 48% risk of recurrence [9]. Mothers with a history of GDM may have disorders of glucose metabolism, as reported in a study on the future risk of type 2 diabetes mellitus [10]. Therefore, clinicians should be aware of the risk of hypoglycaemia in the offspring of women with a history of GDM, even if there was no GDM during the present pregnancy. According to the guidelines of the Japan Society of Obstetrics and Gynecology [11], pregnant women must undergo a 75-g GCT after a positive 50-g GCT in the second trimester (timed glucose ≥ 140 mg/dL at 1 h) or after a casual blood glucose test result ≥ 100 mg/dL. In the present case, the medical records showed that the mother had barely missed the threshold (140 mg/dL) for taking the 75-g GCT because her 1-h 50-g GCT result was 133 mg/dL. This suggests that the mother had a higher serum glucose level than mothers whose timed glucose levels were much lower than the threshold, indicating that her infant might have had an increased risk for hyperinsulinemic hypoglycaemia shortly after birth. We posit that the blood glucose data from prenatal screening or results from GDM testing could help predict the risk of neonatal outcomes. High glucose levels in mothers might cause hyperinsulinemic hypoglycaemia in their infants, even though the mothers do not necessarily meet the GDM diagnostic criteria.

Since worldwide screening has not been established, the frequency of GDM varies widely depending on the guidelines used [1, 12]. The present case suggests that maternal and neonatal outcomes should be considered when determining GDM diagnostic criteria. Indeed, the incidence of neonatal hypoglycaemia varies between cases diagnosed using one-step and two-step screening [1]. Collecting information on the suspected maternal risk factors, including a history of GDM in combination with current serum blood glucose levels, may help predict neonatal hypoglycaemia in infants. The symptoms of hypoglycaemia are not specific [13], making it difficult to differentiate it from other diseases. Because it is associated with severe neonatal sequelae, the prediction of risk based on maternal medical history would be impactful for clinical decision-making and improving neonatal outcomes.

For treatment of symptomatic hypoglycaemia, the recommendations are to immediately resolve the condition because it reflects ongoing brain dysfunction due to glucose deficits [6, 7]. In addition, the severity of hypoglycaemia correlates with the neurodevelopmental sequelae, and delayed diagnosis and treatment after the appearance of symptoms correlate with severe developmental deficits [4]. Symptomatic hypoglycaemia is associated with poor neurodevelopmental outcomes, including cerebral palsy, visual impairment, developmental delay, and brain injuries with abnormal MRI findings [3]. Since serum blood glucose level does not correlate with symptoms, more than half of infants with hypoglycaemia are asymptomatic [14]. Asymptomatic hypoglycaemia has been associated with adverse neurodevelopmental effects [2]. As the present case illustrates, with the present management guidelines, some of these cases might be missed and, therefore, go untreated. We recommend that following the current GDM testing guidelines, clinicians should recognize the risk of hypoglycaemia among neonates without a maternal diagnosis of GDM.

Our clinical approach was successful in treating symptomatic hypoglycaemia without relapse. The AAP and PES guidelines recommend that infants with symptomatic hypoglycaemia should receive a rapid glucose infusion to avoid adverse neurodevelopmental effects [6, 7]. The present case indicates that oral feeding may be effective even in cases of symptomatic hypoglycaemia when rapid glucose infusion is difficult. Feeding can be a viable option for treating neonatal hypoglycaemia while preparing for glucose infusion, especially in facilities where the intravenous approach may be delayed. Buccal dextrose gel is a useful choice for hypoglycaemia in newborns; it has no risk of aspiration and contains no non-human proteins that disturb exclusive breastfeeding. It is increasingly being used for neonatal hypoglycaemia without causing severe adverse effects [15] or interfering with subsequent feeding [16]. It has not always been stocked in the neonatal wards of all facilities, and in such cases, oral maternal or formula milk feeding is one of the options, given its convenience and aveilability. The AAP guidelines also endorse feeding within the first hour of life in infants at risk of hypoglycaemia, including those whose mothers have diabetes [7]. This does not mean that feeding should be preferred to glucose infusion in all cases. Intravenous treatment is still useful among infants with symptoms such as lethargy, hypotonia, poor sucking, and seizure, all of which hinder feeding.

Our case has some limitations. First, this is a clinical report of only one case. However, the present case provided us with an opportunity to share the risk of neonatal hypoglycaemia in neonates born to mothers without current GDM. Accumulating evidence reveals similar cases. Reconsidering the diagnostic criteria of GDM will help elucidate the perinatal outcomes of mothers with glucose metabolic disorders and reduce the number of overlooked neonatal cases. Second, the observation period of the infant after the onset of hypoglycaemia was relatively short, although she was doing well without neurodevelopmental delay at her 6-month check-up. Third, other risk factors of neonatal hypoglycaemia may be overlooked. As no worldwide screening protocol for neonatal hypoglycaemia has been established, we used the screening criteria of our facility, focusing mainly on birthweight, small/large-for-gestational-age, preterm birth, and maternal GDM. However, glucose supply to the mother during delivery and the inaccurate estimation of gestational age are possibly associated with hypoglycaemia development. Previously, intrapartum intravenous glucose infusion was reported to be associated with hypoglycaemia in newborns [17], while another study reported that saline solution boosted with 5% glucose did not increase neonatal hypoglycaemia as well as lactated Ringer’s solution [18]. The infusion of 5% glucose did not probably raise the risk in this case; however, we must be careful about the contents of the intravenous infusions administered to mothers during delivery. The baby’s weight, barely categorized as within normal limits, was also a possible risk factor. Clinicians should be aware of such risk factors, including a maternal history of GDM, when examining medical records.

The present case cautions us regarding the increased risk of transient hyperinsulinemic hypoglycaemia among neonates of mothers with a previous history of GDM, even those who were not diagnosed with GDM during the current pregnancy. Physicians should be aware of the symptoms of hypoglycaemia in infants, regardless of the presence or absence of known risks. The diagnostic criteria for GDM should predict both maternal and neonatal outcomes. Early oral feeding can increase blood glucose levels and improve symptoms, even in infants with severe symptomatic hypoglycaemia.

## Data Availability

All data generated or analysed during this study are included in this published article.

## Abbreviations

GDM: Gestational diabetes mellitus
PES: The Pediatric Endocrine Society
AAP: The American Academy of Pediatrics
GCT: Glucose challenge test
SD: Standard deviations
